# Case Report: Genetic analysis and anesthetic management of a child with Niemann-Pick disease Type A

**DOI:** 10.12688/f1000research.7470.1

**Published:** 2015-12-10

**Authors:** Priti G. Dalal, Melissa Coleman, Meagan Horst, Dorothy Rocourt, Roger L. Ladda, Piotr K. Janicki

**Affiliations:** 1 Department of Anesthesiology, Penn State Hershey Medical Center, Penn State Hershey Children’s Hospital, Hershey, PA, 17033, USA; 2 Department of Surgery, Division of Pediatric Surgery, Penn State Hershey Children’s Hospital, Hershey, PA, 17033, USA; 3 Department of Pediatrics, Department of Pediatrics, Division of Human Genetics, Penn State Hershey Children’s Hospital, Hershey, PA, 17033, USA

**Keywords:** Anesthesia, Niemann-Pick disease, genomic and biochemical analysis

## Abstract

A 14-month-old child, recently diagnosed with Niemann-Pick disease type A, presented for a laparoscopic placement of a gastrostomy tube under general anesthesia. The disease was confirmed and further characterized by genetic testing, which revealed evidence of the presence of two known pathogenic mutations in the *SMPD1* gene, and enzyme studies showed a corresponding very low level of enzymatic activity of acidic sphingomyelinase. The anesthetic management involved strategies to manage an anticipated difficult intubation and avoid post-operative ventilation.

## Introduction

Niemann-Pick disease (NPD) is a rare inherited autosomal recessive lysosomal storage disorder (incidence about 1:40,000 in general population) caused by pathogenic mutations in the *SMPD1* gene and characterised by enzyme studies showing a corresponding very low level of enzymatic activity of acidic sphingomyelinase (ASM), associated with intracellular accumulation of lipids ^1– 3^. People with NPD type A (NPA; generally a very rare presentation of NPD) have little or no ASM production (less than 1% of normal) while those with NPD type B (the most frequent presentation of NPD) have approximately 10% of the normal level of ASM. There is no information on the anesthetic management of a patient with fully genetically characterized and biochemically confirmed NPA, and only one previous report describing a pediatric patient with presumably diagnosed NPA ^4^. In this case report, we describe the genetic background, pathophysiology and anesthetic-related problems in a patient with NPA who presented for surgery.

## Case report

A 14-month-old Caucasian child (residing in the United States, diagnosed with NPA, presented to our hospital pre-anesthesia assessment clinic for laparoscopic placement of a gastrostomy tube. There was no known family member or relatives diagnosed with NPD; mother reported a distant relative of Jewish origin. On physical exam, he was a well-proportioned child, weight was 8.32 kg (10 ^th^ percentile) and height was 77cm (50–75 ^th^ percentile), head circumference was 48.3 cm (90–95 ^th^ percentile). Craniofacial features included frontal bossing, protuberant tongue and mild bilateral ptosis. Generalized hypotonia with head lag and weakness in upper girdle and lower leg muscles, decreased deep tendon reflexes and hepatosplenomegaly (3 cm below the costal margin) were noted. Liver function tests revealed elevation of alanine transaminase (ALT) levels - 90 uts/L (normal range 13–69), aspartate transaminase (AST) levels - 271 uts/L (normal range 9–80), alkaline phosphatase levels - 252 uts/L (normal 8–240) and creatine phosphokinase (CPK) - <20 uts/L (normal range 55–170).

**Figure 1.**
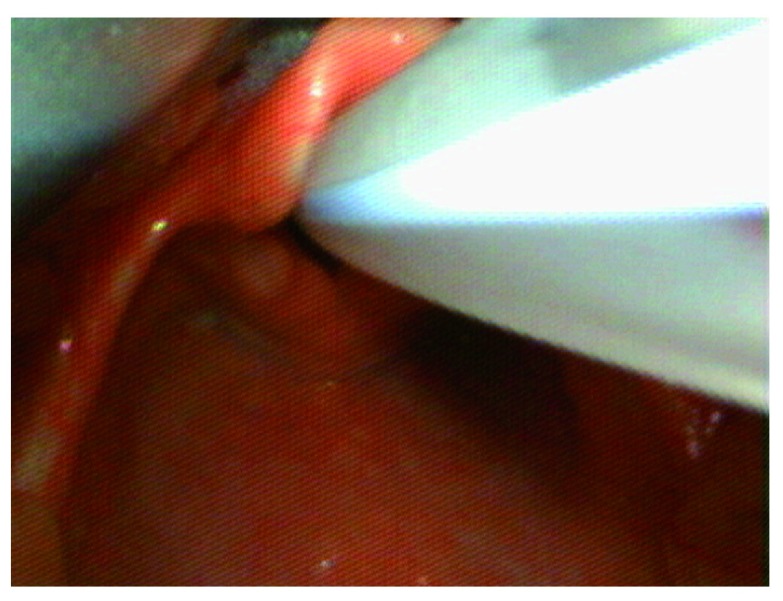
Laryngeal view at intubation with the videolaryngoscope.

The child was scheduled as the first case on the day of surgery. General anesthesia was induced with a 50:50 mixture of nitrous oxide: oxygen and sevoflurane. Following a smooth inhalational induction, intravenous access was established and a single dose of propofol 2 mg/kg was administered intravenously to facilitate tracheal intubation. Tracheal intubation was performed under deep anesthesia with a 4 mm internal diameter cuffed endotracheal tube with a c-MAC videolaryngoscope blade (Cormack and Lehane grade 2 view, Figure 1). Anesthesia was maintained with air: oxygen and sevoflurane mixture (Minimum Alveolar Concentration i.e. MAC=1). A single dose of per rectal acetaminophen 240 mg (30–40 mg/kg) and local anesthetic infiltration with 0.25% bupivacaine were used to facilitate analgesia. Tracheal extubation was uneventful. The child was transported to the post-anesthesia care unit and made an uneventful post-operative recovery.

*Follow-up* The child had G-tube feeds commenced following surgery and subsequently discharged to home the following day and made an uneventful clinical recovery. Subsequently over the course of next few months, the child had episodes of intractable seizures which were medically managed. The patient was referred to home hospice care and expired at home at the age of 2 years.

### Biochemical evaluation

The results of enzymatic evaluation of patient blood and leukocytes by an outside laboratory are presented in Table 1.

**Table 1.** Biochemical enzymatic pattern of investigated patient (by Lysosomal Diseases Testing Laboratory, Philadelphia, PA).

Enzyme	Tissue source	Results (nmol/hr/mg protein)	Comments
Beta-galactosidase	L/P	62.5/17.7	Normal range
Beta-mannosidase	L	100.9	Normal range
Alpha-L-fucosidase	L	52.3	Normal range
Alpha-mannonidase	L	207.1	Normal range
Beta-glucuronidase	L	313.6	Normal range
Beta-hexaminidase A	L	284.0	Normal range
**Sphingomyelinase**	**L**	**0.06**	**Abnormally** **low**
Glucorerebrosidase	L	19.5	Normal range
Alpha-L-idorinidase	L	31.8	Normal range
Alpha-glucosaminidase	P	71.1	Normal range
Mucolipidoses II/III	P	N/A	Ruled out

P – Plasma, L – Leukocytes – **Bold face denotes abnormal results**

### Genetic evaluation

Extracted DNA was PCR-amplified for analysis of the coding exons 1 to 6 of the *SPMD1* gene and their flanking splice sites, using a standard Sanger sequencing approach. Bi-directional sequence was obtained and the DNA sequence was analyzed and compared to the published gene sequence. Reportable variants were confirmed by repeat sequence analysis. Based on the genetic diagnostic laboratory (Proprietary information from GeneDx, Gaithersburg, MD) 99% sensitivity is expected in detecting mutations identifiable by sequencing. Please refer to Table 2.

**Table 2.** The sequencing analysis provided evidence of the presence of two pathogenic mutations in the *SMPD1 gene*.

Gene	cDNA	Variant	Zygosity	Classification
SPMD1	*c.573delT	p.Ser192AlafsX65	heterozygous	Disease-causing mutation
SPMD1	**c.1783_1784delCT	p.Ala597ProfsX7	heterozygous	Disease-causing mutation

*The normal sequence with the base that is deleted in braces is: ACCCCC(T)AGCC. **The normal sequence with the bases that are deleted in braces is: TACT(CT)TTGT

## Discussion

Our anesthesia management strategy focused on avoiding muscle relaxants and narcotic analgesics. Hence, endotracheal intubation under deep anesthesia without muscle paralysis and non-opioid analgesics, i.e., acetaminophen, and infiltration of the surgical wound with local anesthetic were contemplated. Adequate pain control was possible with this technique since the procedure was performed laparoscopically. Although the child had mild elevation of liver enzymes, acetaminophen was preferred as a short-term analgesic. Use of atracurium, midazolam, sevoflurane and fentanyl has been described in the management of anesthesia in a 2-year-old patient with presumable NPA presenting for emergency splenectomy ^4^. The child had severe hepatosplenomegaly, ventilation improved after splenectomy and the child required a post-operative ICU stay for 9 days.

The observed c.573delT mutation in the *SMPD1* gene in our case has been reported previously in association with NPD type A ^5^. The deletion causes a frame shift starting with codon Serine 192, changes this amino acid to an Alanine residue and creates a premature Stop codon at position 65 in the new reading frame denoted p.Ser192AlafsX65. This mutation is predicted to cause abnormal protein function either through protein truncation or nonsense-mediated mRNA decay. The c1783_1784delCT deletion causes a frame shift starting with codon Alanine 597, changes this amino acid to a Proline residue, and creates a premature stop codon at position 7 of the new reading frame denoted p.Ala597ProfsX7. This mutation is predicted to cause loss of normal protein function through protein truncation. Specifically, the last 35 correct residues are replaced by six incorrect residues. Therefore, the presence of these mutations is consistent with the diagnosis of an *SMPD1*-related disorder, if the mutations were inherited on different alleles (in trans), i.e., each mutation coming from a different parent ^6^. This result therefore permits mutation-specific carrier testing for family members and prenatal diagnosis for the parents of this child, if desired. However, targeted carrier testing of both parents is necessary prior to or concurrently with any carrier testing or predictive testing in this family.

Patients with NPA and NPB have significantly different clinical course. While NPA is associated with severe neurological deficits leading to early death, patients with NPB have minimal neurological involvement and may survive to adulthood, although hepatosplenomegaly and cardiorespiratory problems may occur. We believe therefore that it is important to distinguish these form of NPD preoperatively, because NPA requires much more strict anesthetic management (avoidance of muscle relaxants and opioids, if at all possible) and it could associated more frequently with step-up level of postoperative care.

## Consent

Written informed consent for publication of clinical details and/or images was obtained from the parent of the patient.
