# Supplementary material for: Case Report: Genetic analysis and anesthetic management of a child with Niemann-Pick disease Type A
Source: F1000Res. 2015 Dec 10;4:1423. [Version 1] doi: 10.12688/f1000research.7470.1 (PMC4743149; doi:10.12688/f1000research.7470.1)
Supplement: Peer review history

# Peer review: Referee response for version 1

**DOI:** 10.5256/f1000research.8048.r12222

**Published:** 2016-02-01

**Authors:** Rakesh Garg

**Affiliations:** 1 Department of Anaesthesiology, Pain Medicine and Critical Care, All India Institute of Medical Sciences, New Delhi, Delhi, India

Please provide more details of airway management i.e. difficulty level. Was it predicted and preparation done before inducing anaesthesia?

Please provide detail (positive or negative ) for any other additional comorbidities - structural or functional.

Please mention duration of surgery, need of any NMB drugs, other analgesia, hemodynamic status intraoperatively.

Since the title says anaesthetic management as well, It would be good if authors could add an table for various perioperative concerns in such child and their suggestions for the management.

I have read this submission. I believe that I have an appropriate level of expertise to confirm that it is of an acceptable scientific standard.

# Peer review: Referee response for version 1

**DOI:** 10.5256/f1000research.8048.r11964

**Published:** 2016-02-01

**Authors:** James J. Fehr

**Affiliations:** 1 St Louis Children's Hospital, Washington University in St. Louis, St Louis, MO, USA

This is novel case report of a 14 month old with NPA who is undergoing an anesthetic. The manuscript is generally written, a few missing articles were noted, but the substance of the article is sound. The report is supported by appropriate references from the literature and the case description is succinct but complete. A modest improvement might be made by including the clinical presentation of NPA (what to be concerned about) in the introduction which would serve to focus the reader on the subsequent anesthetic management.

I have read this submission. I believe that I have an appropriate level of expertise to confirm that it is of an acceptable scientific standard.
